# Adult hypertrophic pyloric stenosis that improved by spontaneous double channel pylorus formation

**DOI:** 10.1002/jgh3.12458

**Published:** 2020-12-02

**Authors:** Aiji Hattori, Hiroyuki Kawabata, Yuhei Umeda, Junya Tsuboi, Reiko Yamada, Yasuhiko Hamada, Kyosuke Tanaka

**Affiliations:** ^1^ Department of Endoscopy Mie University Hospital Tsu Japan; ^2^ Department of Gastroenterology and Hepatology Mie University Hospital Tsu Japan; ^3^ Department of Gastroenterology Saiseikai Matsusaka General Hospital Matsusaka Japan

**Keywords:** adult, endoscopy, endosonography, peptic ulcer perforation, pyloric stenosis, hypertrophic

## Abstract

Adult hypertrophic pyloric stenosis (AHPS) is a rare disease and presents as pyloric obstruction. Double pylorus is also a rare condition due to a gastroduodenal fistula connecting from the gastric antrum to the duodenum. A 42‐year‐old woman without a history of vomiting in infancy presented with postprandial abdominal distension and repeated vomiting. Abdominal computed tomography showed gastric dilatation and wall thickening of the distal stomach. Endoscopy and contrast gastrography revealed gastric outlet obstruction due to stenosis and an ulcer in the antral and pyloric region. Endoscopic ultrasonography revealed circumferential thickening of the muscularis propria layer of the pylorus. Her symptoms improved with treatment consisting of drainage, fasting, and a proton pump inhibitor. Two weeks after onset, follow‐up endoscopy revealed a healing ulcer and double channel pylorus. Based on her clinical course and findings of clinical images, she was diagnosed with gastric outlet obstruction due to AHPS that was improved by double channel pylorus formation. In conclusion, AHPS that was improved by double channel pylorus formation is an extremely rare condition, and we should be aware of this disease entity.

## Graphical abstract

This article presents an adult‐onset case of hypertrophic pyloric stenosis (AHPS). In this case, long term (over six months) persisting thickening of the muscularis propria in the pyloric region proved the precise diagnosis of AHPS. Furthermore, it is very interesting that double pylorus due to possible penetrating gastric ulcer led to the improvement of her symptom.

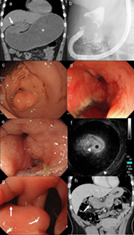

## Introduction

Adult hypertrophic pyloric stenosis (AHPS) is a rare disease and presents in adult life as pyloric obstruction. It is a benign disease resulting from hypertrophy of the circular fibers of the pyloric canal. The most common type of AHPS is due to peptic ulcer disease, malignancy, and certain inflammatory diseases.^1^ However, it is usually difficult to make the diagnosis of AHPS due to the lack of specificity of clinical and laboratory abnormalities; therefore, surgical resection is often performed to establish the diagnosis and provide treatment.^2^ Here, we report a rare case of AHPS in an adult woman with double pylorus formation which led to improvement of her symptoms.

## Case report

**Figure 1:**
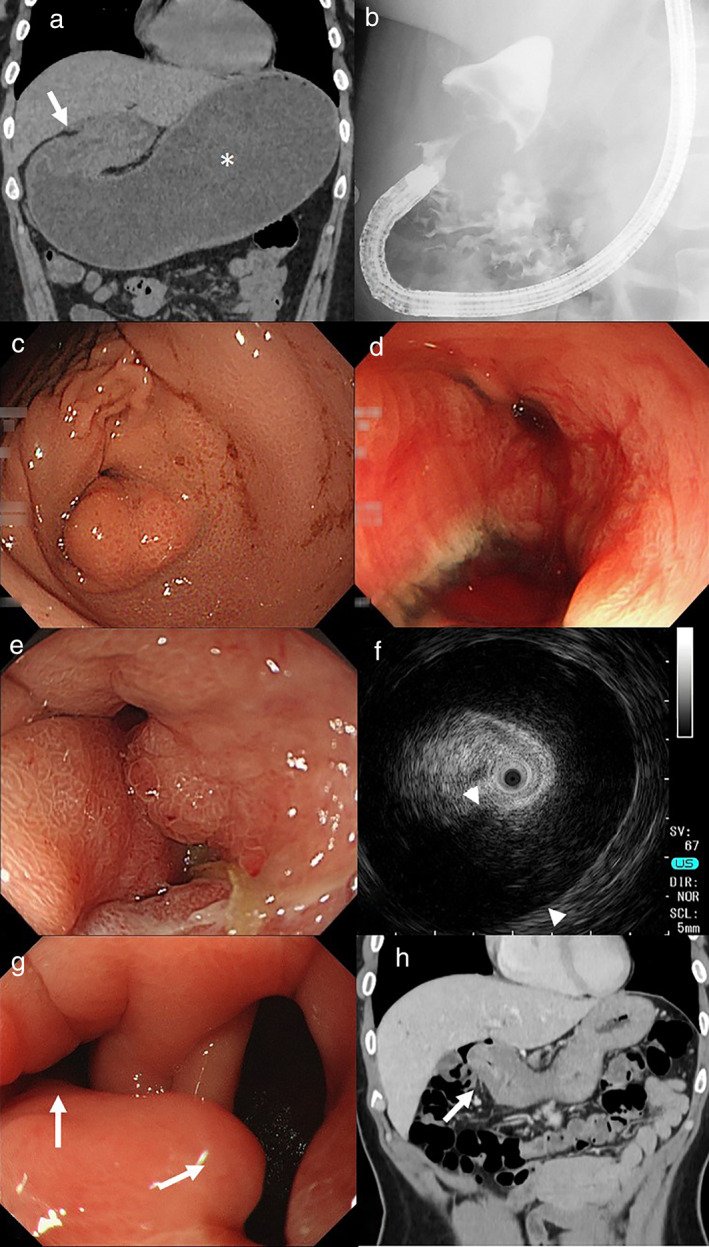
Images of adult hypertrophic pyloric stenosis in our patient. (a) Abdominal CT obtained on the day the patient came to the emergency department (hospital day 1) showed gastric dilatation (asterisk) and wall thickening of the distal stomach (arrow). (b) Contrast gastrography on hospital day 1 showed a stenotic pylorus. (c) Endoscopy on hospital day 1 revealed gastric stenosis in the pylorus. (d) Endoscopy on hospital day 1 showed that there was a deep ulcer in the prepyloric region. (e) Endoscopy performed on hospital day 12 showed a healing ulcer in the prepyloric region. (f) Endoscopic ultrasonography performed on hospital day 12 showed circumferential thickening of the muscularis propria layer (arrowheads) of the pylorus. (g) Endoscopy performed two months after presentation to our emergency room revealed a double channel pylorus (arrows). (h) Abdominal CT performed six months after presentation to our emergency room showed residual wall thickening of the distal stomach (arrow).

A 42‐year‐old woman without a history of vomiting in infancy presented to the emergency department with postprandial abdominal distension and repeated vomiting but no abdominal pain. Laboratory examination showed normal results, including a normal serum Helicobacter pylori antibody level, except for mild anemia. Abdominal computed tomography (CT) showed gastric dilatation and wall thickening of the distal stomach (Fig. 1a, asterisk, arrow). Endoscopy and contrast gastrography revealed gastric outlet obstruction due to stenosis of the antral and pyloric region (Fig. 1b,c), and a deep ulcer in the stenotic antrum (Fig. 1d). She was admitted to our hospital and treated with nasogastric tube drainage, fasting, and administration of a proton pump inhibitor. Three days later, her symptoms improved and she could start to take a liquid diet. Ten days after the initiation of treatment, she was asymptomatic and could eat a normal diet. Follow‐up endoscopy performed on hospital day 12 showed a healing ulcer in the prepyloric region (Fig. 1e) and a distinct opening to the duodenum at the edge of the healing ulcer that was different from the original pylorus. Endoscopic ultrasonography (EUS) revealed circumferential thickening of the muscularis propria layer of the pylorus with a maximum thickness of over 10 mm (Fig. 1f, arrowheads). Pathological examination of multiple biopsy specimens taken from the ulcer and surrounding mucosa showed no evidence of malignancy. Since the patient was asymptomatic, she was discharged from the hospital on hospital day 21. Additional follow‐up endoscopic examinations revealed a clear double pylorus (Fig. 1g, arrows). The endoscope could easily pass through both channels to the duodenum. Based on her clinical course and findings of clinical images, she was diagnosed as having gastric outlet obstruction due to AHPS that was improved by spontaneous double channel pylorus formation. Six months after onset, CT showed residual wall thickening of the distal stomach without gastric dilatation (Fig. 1h, arrow) and EUS revealed residual circumferential thickening of the muscularis propria layer of the pylorus, indicating that she still had AHPS although she did not have any abdominal symptoms.

Our patient's clinical and imaging findings suggested that the double pylorus formed in our patient in the following manner. When we retrospectively reviewed the endoscopic images from the EGD examination that the patient had undergone 1 year prior to the current admission, antral narrowing was observed. She may have had asymptomatic AHPS at that time. When the patient came to the emergency room, endoscopy and contrast gastrography revealed gastric outlet obstruction due to stenosis and a deep ulcer in the stenotic antrum. During the hospital admission, the deep ulcer made a fistula with the duodenum that would eventually become a double pylorus. After double pylorus formation, the patient no longer had symptoms of AHPS and she was discharged from the hospital.

## Discussion

AHPS can be classified into the following three types. The first type is the late stage of infantile hypertrophic pyloric stenosis (HPS). The second type is HPS commencing in adult life but secondary to other diseases such as hiatal hernia, duodenal ulcer, gastric ulcer, tumors, or inflammatory diseases. The third type is primary idiopathic HPS (IHPS) presenting in adult life without any apparent cause.^1^ Hypertrophy of the pyloric muscle is milder in patients with the second type of AHPS than in patients with the primary types of AHPS, and patients with the second type of AHPS often do not have any symptoms.^1^ The clinical symptoms of adult IHPS are similar to the clinical symptoms of gastric outlet obstruction induced by other causes including epigastralgia, easy satiety, and postprandial nausea or vomiting.^2^ The exact etiology of AHPS is unclear, and it is usually difficult to diagnose AHPS before surgical resection.^2^ In patients with AHPS, gastrography usually shows an elongated and narrow pyloric canal, and delayed gastric emptying due to pyloric stenosis.^2^, ^3^ Abdominal CT often shows thickening of the distal gastric wall in patients with AHPS.^2^ Endoscopic ultrasonography is helpful in diagnosing AHPS. The wall thickness of a normal pyloric muscle varies from 3.8 to 8 mm.^4^ In contrast, the wall thickness of the pyloric muscle in patients with AHPS is often over 10 mm.^5^ In our case, the pyloric canal was elongated and narrow due to the stenotic antral and pyloric region, and the thickness of the distal gastric wall was increased on CT. EUS revealed circumferential thickening of the pyloric muscle with a maximum thickness of more than 10 mm. The diagnosis of AHPS was established according to her clinical and imaging findings.

Patients with AHPS who have persistent symptoms usually undergo surgical treatment such as partial gastrectomy, gastroenterostomy, pyloromyotomy, or pyloroplasty. Recently, endoscopic treatment methods including pyloroplasty^6^ and pyloromyotomy^7^ have been reported. In our case, spontaneous double pylorus formation after conservative treatment avoided these invasive treatments.

On another note, double pylorus due to a gastroduodenal fistula connecting from the gastric antrum to the duodenum is a rare condition.^8^, ^9^ An acquired double pylorus is often caused by a chronic gastric ulcer that penetrates and creates a fistula with the duodenal bulb.^10^ In our case, a gastric ulcer may have been present in the prepyloric region for a short while before the onset of gastric obstruction, because the narrowed antrum that had been observed by endoscopy 1 year previously suggested that the patient had been suffering from asymptomatic AHPS at that time. During the present hospitalization, after gastric ulcer treatment, the pylorus was found to have double channels that connected to the duodenal bulb and the descending duodenum, respectively. The endoscopic examination that had been performed at the onset of gastric outlet obstruction revealed a deep ulcer in the stenotic antrum, and the ulcer might have penetrated through the antral wall and formed a fistula with the descending duodenal. Furthermore, we hypothesized that the double pylorus that formed after the development of gastric ulcer led to improvement of her AHPS symptoms and the patient could avoid surgical treatment.

In conclusion, we presented an extremely rare patient with gastric outlet obstruction due to AHPS whose symptoms improved by double channel pylorus formation. We should be aware of this disease entity and this disease entity should be included in the differential diagnoses of spontaneous improvement of gastric outlet obstruction.
